# Treatment of antibody-mediated rejection in heart transplantation: from empirical combination to multi-target precision intervention

**DOI:** 10.3389/fimmu.2026.1902286

**Published:** 2026-08-03

**Authors:** Ning Xu, Yaqin Dong, Yehong Yue, Liang Zhou

**Affiliations:** 1 Department of General Medicine, The Second Affiliated Hospital of Wannan Medical College, Wuhu, China; 2 Department of Infectious Disease, Shaoyang Central Hospital, Shaoyang, China

**Keywords:** antibody-mediated rejection, complement inhibitor, donor-specific antibody, heart transplantation, IL-6, precision immunosuppression, proteasome inhibitor

## Abstract

Antibody-mediated rejection (AMR) is the primary immunological obstacle limiting long-term survival in heart transplant recipients. Driven by donor-specific antibodies (DSA), AMR damages graft microvasculature through multiple pathways, including complement activation, antibody-dependent cellular cytotoxicity, and sustained endothelial activation. It may present as acute hemodynamic collapse or progress insidiously in a subclinical manner, ultimately culminating in cardiac allograft vasculopathy (CAV) and graft failure. Over the past two decades, with successive updates to the ISHLT pathological diagnostic system, AMR has evolved from a vague clinical concept into a distinct entity defined by explicit histopathological and immunopathological criteria. However, therapeutic advances have markedly lagged behind the deepening understanding of its diagnosis. For a long time, the first−line regimen has consisted of plasma exchange combined with intravenous immunoglobulin (IVIG). Nevertheless, its mechanism—clearance of circulating antibodies plus broad−spectrum immunomodulation—neither targets the source of antibody production (long−lived plasma cells) nor selectively blocks terminal effector pathways. Approximately 30–50% of refractory AMR cases respond poorly to this regimen. Over the past decade, agents targeting plasma cells (proteasome inhibitor bortezomib), the terminal complement component (C5 monoclonal antibody eculizumab), the IL−6 pathway (tocilizumab, clazakizumab), and the more recent anti−CD38 monoclonal antibody (daratumumab) have entered clinical practice, shifting AMR treatment from having no actionable targets to enabling multi−node intervention. This review delineates the rationale underlying this evolution: the treatment paradigm is transitioning from empirical combination therapy to biomarker−driven, multi−target precision intervention covering the entire sequence of “production–circulation–effector pathways.” However, the field remains in a distinct transitional phase characterized by “weapons available but tactics lacking”—the vast majority of evidence derives from single−center, retrospective, small−sample observational studies, with no randomized controlled trial (RCT) specifically designed for AMR in heart transplantation. Consensus is lacking on how to select, combine, and sequence these agents based on individual immune phenotypes. Stratified therapy, dynamic biomarker monitoring, and rational multi−target combination will be the core directions for solving this challenge in the coming decade.

## 1 Introduction

Heart transplantation represents the most effective curative treatment for end-stage heart failure; however, long-term graft survival remains constrained by immune rejection, among which antibody-mediated rejection (AMR) — centered on humoral immunity — is particularly challenging. Epidemiological data indicate that the incidence of clinically significant AMR within the first year after heart transplantation is approximately 10%–15%, and the cumulative detection rate over several years increases further when subclinical cases identified by protocol biopsies are included (1–3). Of concern, a considerable proportion of AMR does not present with typical graft dysfunction but is discovered incidentally during routine endomyocardial biopsy (EMB); such subclinical AMR is also associated with adverse outcomes (3–9).

The impact of AMR on prognosis is multidimensional. First, it directly reduces recipient survival: medium- to long-term survival rates in patients with AMR are significantly lower than those without rejection, particularly in severe cases accompanied by hemodynamic compromise (2, 10). Second, AMR is an independent risk factor for cardiac allograft vasculopathy (CAV) — persistent antibody−mediated endothelial injury is considered a key driver of accelerated diffuse intimal hyperplasia in graft coronary arteries (1, 11, 12). In a retrospective analysis involving more than one thousand patients and over ten thousand biopsy specimens, all grades of pAMR (pAMR1 through pAMR3) were associated with an increased risk of cardiovascular death, with the highest risk observed in severe pAMR3 (13). From a clinical burden perspective, patients with AMR often experience recurrent hospitalizations, intensified immunosuppression, progressive graft dysfunction, and even retransplantation, consuming substantial healthcare resources. Given the chronic shortage of donor hearts, each episode of AMR imposes an intolerable cost (2, 10, 14–19).

Our understanding of AMR has been progressively refined through iterative revisions of diagnostic criteria. In the early period (1990 ISHLT working formulation), diagnosis relied mainly on descriptive features such as “immunofluorescence positivity, vasculitis, or severe edema in the absence of cellular infiltration,” which were vague and poorly reproducible (20, 21). The 2005 working formulation for the first time incorporated clinical dysfunction, serological DSA, and histologic changes as parallel diagnostic elements. The 2010–2011 consensus conference introduced a pivotal shift — redefining AMR as a diagnosis made by the pathologist, no longer requiring concurrent clinical dysfunction or positive serum DSA, thereby enabling systematic identification of asymptomatic AMR (3, 21–24).

The 2013 ISHLT “Consensus on Naming of Antibody-Mediated Rejection in Heart Transplantation — Pathological Diagnostic Working Formulation” further operationalized this concept into an actionable pAMR grading system: pAMR0 (negative), pAMR1(h) (histopathological changes only), pAMR1(i+) (immunopathological changes only), pAMR2 (both positive), and pAMR3 (severe) (21). One point warrants clarification: in this system, immunopathological staining for C4d and other markers remains a core component — the designation of pAMR1(i+) depends precisely on it. What has been “de−mandated” is clinical dysfunction and serological DSA, not C4d. This consensus also explicitly recognized a phenotype of AMR that is C4d−negative but exhibits morphological microvascular inflammation and macrophage infiltration, indicating that complement−independent injury pathways should not be overlooked (21, 25). The current complete diagnostic approach requires integration of three levels of information: histopathology, immunopathology, and serology (3, 21, 26–29).

The refinement in diagnostic understanding imposes two new requirements for therapy. First, earlier identification of subclinical AMR to enable intervention before irreversible damage occurs. Second, distinguishing active AMR from chronic AMR to align treatment intensity accordingly (30–34). Thus, this review will sequentially elaborate on the central theme of “how empirical combination therapy is transitioning toward multi−target precision intervention.”

## 2 Mechanistic basis of therapeutic targets: an overview of AMR pathophysiology

**Figure 1:**
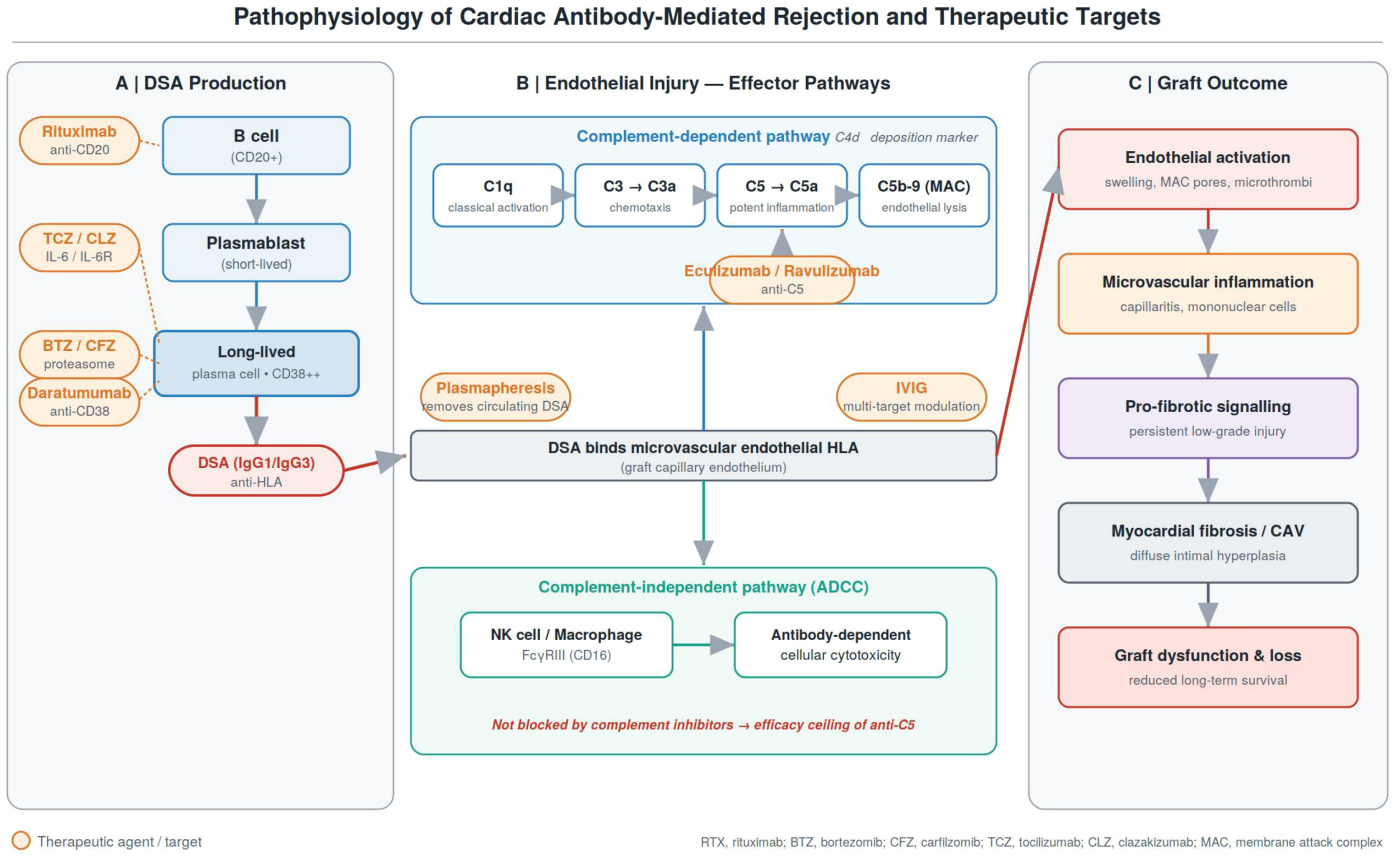
Pathophysiology of cardiac AMR and therapeutic targets. **(A)** DSA production axis (B cell to plasmablast to long-lived plasma cell) with sites of action of rituximab (anti-CD20), bortezomib/carfilzomib (proteasome), daratumumab (anti-CD38) and tocilizumab/clazakizumab (IL-6/IL-6R). **(B)** Effector pathways: the complement-dependent cascade (C1q to C3/C3a to C5/C5a to C5b-9/MAC; site of eculizumab/ravulizumab) and the complement-independent pathway (ADCC via NK cells and macrophages through FcgammaRIII), which is not blocked by complement inhibitors and defines the efficacy ceiling of anti-C5. Plasmapheresis removes circulating DSA; IVIG acts at multiple points. **(C)** Graft outcome: endothelial activation to microvascular inflammation to fibrosis/CAV to graft loss.

Understanding the treatment of AMR requires a prior understanding of the complete chain from DSA “production” to “pathogenicity,” as existing and investigational drugs act on different links of this chain, respectively (**Figure 1**) (35).

### 2.1 DSA production and the central role of plasma cells

Sensitized recipients (due to prior pregnancy, blood transfusion, mechanical circulatory support device implantation, or previous transplantation) may carry pre−existing anti−HLA antibodies. After transplantation, recipient B cells can also recognize donor HLA antigens and, upon co−stimulation by helper T cells, differentiate into two types of antibody−secreting cells (36, 37). One type comprises short−lived plasmablasts, responsible for the rapid surge of antibodies during the acute phase. The other type consists of long−lived plasma cells that home to the bone marrow microenvironment and can survive for months to years, continuously producing DSA at a low rate and forming a “reservoir” for recurrent antibody rebound (37, 38). This distinction is crucial: it explains why simply clearing circulating antibodies (plasma exchange) achieves only transient effects, and why rituximab, which depletes CD20+ B cells, fails to eliminate the source of antibodies — because terminally differentiated long−lived plasma cells no longer express CD20 (38–40). Key molecular nodes directly relevant to therapy include CD20 (target of rituximab), CD38 (target of daratumumab), the proteasome (target of bortezomib), and the IL−6/IL−6R signaling axis (target of tocilizumab and clazakizumab) (26, 31, 39–46).

### 2.2 Complement−dependent injury pathway

After DSA (with IgG1 and IgG3 subclasses having the strongest complement−fixing capacity) binds to HLA antigens on the microvascular endothelial surface of the graft, its Fc segment recruits C1q, initiating the classical complement pathway (30, 47). The cascade sequentially generates C3 convertase (which cleaves C3 to produce the chemotactically active C3a) and C5 convertase (which cleaves C5 to produce the potent inflammatory mediator C5a and the precursor of the membrane attack complex, C5b), ultimately forming the C5b−9 membrane attack complex (MAC) (47, 48). MAC perforates the endothelial cell membrane, leading to endothelial swelling, lysis, and basement membrane exposure, which subsequently activates coagulation and platelets, resulting in microthrombi formation (30, 48). C4d represents the “molecular footprint” left by this process and is a classic marker for the immunopathological diagnosis of AMR (21, 49). This terminal complement step is precisely the site of action of eculizumab (anti−C5) (41, 50–54).

### 2.3 Complement−independent injury pathway

Complement is not the sole pathway of AMR injury. The Fc segment of endothelium−bound DSA can be recognized by FcγRIII (CD16) on the surface of natural killer (NK) cells and macrophages, triggering antibody−dependent cell−mediated cytotoxicity (ADCC) (55, 56). Infiltration of NK cells and macrophages within the microvascular lumen is an important pathological feature of AMR and can occur without prominent C4d deposition — this constitutes the molecular basis of “C4d−negative AMR” (25, 56, 57). From a therapeutic perspective, the existence of ADCC reveals a critical ceiling of efficacy: any strategy that merely blocks complement cannot reach this pathway, mechanistically limiting the upper bound of efficacy of C5 inhibitors (41, 56). Furthermore, DSA binding to endothelium per se can directly activate endothelial cells via “inside−out” signaling, upregulating adhesion molecules and promoting proliferation (58–69).

### 2.4 Chronic outcome: microvascular inflammation and myocardial fibrosis

When DSA persists, injury recurs repeatedly, and the intensity is insufficient to cause acute decompensation, the graft enters the chronic AMR phase. Persistent low−grade endothelial activation drives the secretion of pro−fibrotic factors, multilayering of the microvascular basement membrane, interstitial fibrosis, and microvascular rarefaction. Clinically, this manifests as insidiously progressive graft dysfunction and interacts reciprocally with diffuse intimal hyperplasia of CAV (11, 12, 70). The core therapeutic dilemma at this stage is that once fibrosis and vascular remodeling are established, they are difficult to reverse. Existing targeted agents mostly act on the “production” and “effector” links and have limited efficacy against established structural lesions. This is precisely the fundamental reason, emphasized repeatedly later in this review, for the value of early and subclinical−stage intervention (32, 70–77).

## 3 Conventional therapy: cornerstones and unmet needs

Before the advent of targeted agents, the management of AMR was built on two pillars: “antibody clearance plus broad−spectrum immunomodulation.” These approaches remain the backbone of most regimens to date, but their limitations precisely define the gaps that subsequent targeted therapies have sought to fill.

### 3.1 Plasma exchange: first−line clearance of circulating antibodies

Plasma exchange removes plasma by centrifugation or membrane separation, non−selectively clearing circulating immunoglobulins and some complement components. It is the most direct physical means of rapidly reducing the DSA load (10, 78). A course of several sessions reduces the mean fluorescence intensity (MFI) of DSA by approximately 25–40%, the exact reduction depending on HLA class and on baseline antibody strength; a single exchange of one plasma volume removes a substantial fraction of intravascular IgG, but re-equilibration limits the net inter-session decrement (79). In acute severe AMR with hemodynamic compromise, it is often used as a first−line intervention to buy time (10, 78, 80). However, its limitations are equally clear: because it does not target the source of antibody production, most patients experience DSA rebound within 48–72 hours after cessation of treatment (38, 80). Therefore, plasma exchange is typically administered every other day for a total of 3–5 sessions in clinical practice and must be combined with immunomodulatory or anti−plasma cell agents (10, 80–82).

### 3.2 Intravenous immunoglobulin: multi−mechanism basal immunomodulation

The mechanisms of action of IVIG are multiple rather than singular: its anti−idiotypic antibodies can neutralize DSA; high−dose IgG competitively occupies the neonatal Fc receptor (FcRn), accelerating the catabolism of endogenous pathogenic antibodies; it also inhibits complement deposition, downregulates FcγR expression on effector cells, and modulates the cytokine network (83, 84). A frequently confused dosing issue exists in clinical practice: high−dose IVIG (approximately 2 g/kg) is used for immunomodulation and constitutes a component of AMR therapy, whereas low−dose IVIG (approximately 100–500 mg/kg) is primarily used for infectious replacement therapy in hypogammaglobulinemia — the purposes and mechanisms differ (83, 85). The limitation of IVIG is that, when used alone, it has limited efficacy against DSA with high MFI and strong complement−fixing capacity, as well as against AMR with overt cardiac dysfunction. Therefore, it is almost always combined with plasma exchange or other agents (10, 80, 83, 86–89).

### 3.3 Rituximab: attempts and reflections on anti−CD20 B−cell depletion

Rituximab depletes peripheral B cells by binding to CD20, theoretically reducing antigen presentation, attenuating memory B−cell responses, and decreasing subsequent antibody production (39, 40, 90). Clinically, it maintains circulating B−cell depletion for 6–12 months (91). However, a fundamental mechanistic limitation restricts its role in AMR: terminally differentiated long−lived plasma cells do not express CD20 and are therefore unaffected by rituximab, continuing to produce DSA (39, 40, 91). Evidence supporting its efficacy is derived primarily from retrospective cohorts and combination regimens, suggesting that adding rituximab to plasma exchange/IVIG may improve DSA clearance rates. However, high−quality RCTs specifically targeting AMR in heart transplantation are lacking, and previous randomized studies in different organs have yielded inconsistent results (10, 90, 92). Consequently, current major guidelines and position statements no longer list rituximab as a first−line core recommendation for AMR, instead largely restricting its use to cases with concomitant cellular rejection or as an adjunctive agent (26, 30, 31, 93–96).

Summary of this section: Conventional regimens have established an irreplaceable cornerstone in “clearing circulating antibodies” and “providing broad−spectrum immunomodulation.” However, they neither target the source of antibody production (long−lived plasma cells) nor selectively block terminal effector pathways (complement, ADCC), thereby leaving a substantial unmet need for refractory and recurrent AMR.

## 4 Breakthroughs in novel targeted therapies

The core concept of targeted therapy over the past decade has been to sequentially intercept the DSA “production–circulation–effector” chain. A premise that runs throughout must be stated upfront: to date, no targeted agent has been evaluated in a randomized controlled trial specifically for AMR in heart transplantation. The majority of evidence cited in this section is derived from single−center case series and retrospective cohorts, with some mechanistic insights and efficacy inferences extrapolated from the kidney transplantation literature (31, 41, 97–100).

Before reviewing the individual agents, we make explicit a distinction that is often blurred in the AMR literature and that determines how the evidence below should be read. Three clinical settings are involved, and evidence generated in one does not transfer automatically to the others: (i) desensitization, in which pre-formed DSA are lowered in a sensitized candidate before transplantation; (ii) perioperative prophylaxis, in which early complement-mediated injury is blocked at the time of transplantation; and (iii) treatment of established AMR, in which active injury is halted in a functioning allograft. In the sections that follow we therefore state, for each key study, which of these settings it addresses; for example, the pivotal bortezomib reports of Woodle and Sberro-Soussan and the eculizumab trials of Stegall and Marks are desensitization or perioperative-prophylaxis studies, and the Vo tocilizumab report is a desensitization study, none of which directly tests the treatment of established cardiac AMR. A second caveat concerns the organ of origin. Most higher-level evidence comes from kidney transplantation, and its extrapolation to the heart is not straightforward: the two organs differ in the endpoints available (there is no cardiac surrogate equivalent to the eGFR slope used in renal trials), in the hemodynamic urgency of acute rejection, and in biopsy sampling and grading (the ISHLT pAMR scheme versus the Banff classification). We consequently indicate the source organ of the principal studies and treat kidney-derived conclusions as hypothesis-generating rather than confirmatory for the cardiac setting (**Table 1**).

**Table 1:** Traditional and novel agents for cardiac antibody-mediated rejection: targets, evidence and positioning.

Agent	Primary target	Representative study (design; n)	Key efficacy signal	Main adverse effects	Guideline/positioning	Evidence level
Plasmapheresis	Circulating antibody (non-selective)	Multiple case series	Rapid DSA/MFI reduction; rebound in 48–72 h without adjunct	Catheter/coagulopathy, hypotension, infection	First-line backbone (acute AMR)	Low (observational)
IVIG (high-dose 2 g/kg)	Multi-target (FcRn, complement, idiotype)	Case series; adjunct in most cohorts	Adjunctive DSA modulation	Volume load, headache, thrombosis, renal	First-line backbone (with PLEX)	Low–moderate
Rituximab	CD20 (B cells)	Zarkhin RCT (kidney); cardiac case series	B-cell depletion 6–12 mo; does not target plasma cells	Infusion reaction, infection, late hypogammaglobulinemia, PML (rare)	Adjunct/mixed ACR; no longer core first-line	Low
Bortezomib	26S proteasome (plasma cells)	Everly/Woodle (kidney); Morrow, Patel (heart)	DSA ↓ in a majority of treated patients	Peripheral neuropathy (mostly grade 1–2), thrombocytopenia, GI, infection	Refractory AMR (off-label)	Low (no heart RCT)
Carfilzomib	26S proteasome (irreversible)	Tremblay (kidney desensitization)	DSA reduction; less neuropathy than bortezomib	Cardiopulmonary, cytopenias	Investigational	Low
Eculizumab	Terminal complement C5	Stegall, Marks (kidney RCT); Patel, Kittleson (heart)	Prevents early AMR in sensitized recipients; no effect on DSA	Meningococcal/Neisseria infection (vaccination mandatory), high cost	Prevention in high-risk/rescue (off-label)	Moderate (kidney)/Low (heart)
Tocilizumab	IL-6 receptor	Choi, Vo, Pottebaum (kidney)	DSA modulation; stabilization in chronic AMR	Infection, transaminitis, neutropenia, GI perforation (rare)	Chronic/refractory AMR (off-label)	Low
Clazakizumab	IL-6 (ligand)	Doberer Ph2; IMAGINE Ph3 (kidney)	Ph2 DSA modulation; Ph3 did not meet primary endpoint (terminated)	Infection, cytopenias, GI perforation	Investigational (kidney); negative pivotal trial	Moderate (RCT, negative)
Daratumumab	CD38 (plasma cells, NK)	Heart case series; kidney case series	DSA ↓, dd-cfDNA ↓, pAMR improvement	Cytopenias, infection, infusion reactions, interferes with crossmatch	Refractory AMR (off-label)	Very low (case-level)
Felzartamab	CD38	Mayer Ph2 RCT (kidney late AMR)	Improvement in molecular AMR activity vs placebo	Infusion reactions, infection	FDA Breakthrough Therapy + Orphan Drug Designation (kidney AMR); phase 3 (TRANSCEND) ongoing	Moderate (kidney RCT)

ACR, acute cellular rejection; DSA, donor-specific antibody; GI, gastrointestinal; IVIG, intravenous immunoglobulin; MFI, mean fluorescence intensity; PLEX, plasma exchange; PML, progressive multifocal leukoencephalopathy. No randomized controlled trial of any targeted agent exists specifically in cardiac AMR.

### 4.1 Directly targeting plasma cells: proteasome inhibitors

#### 4.1.1 Mechanism of action

Bortezomib is the first proteasome inhibitor introduced into the transplantation field. It reversibly inhibits the chymotrypsin−like activity of the 26S proteasome, preventing the degradation of misfolded and surplus immunoglobulins within cells. These proteins then accumulate, triggering endoplasmic reticulum stress and the unfolded protein response, ultimately leading to apoptosis (97, 101). Because long−lived plasma cells synthesize and secrete large quantities of immunoglobulins daily, they are highly dependent on proteasome function and are therefore particularly sensitive to proteasome inhibition — this constitutes the mechanistic basis by which bortezomib can reach the “source of antibodies” that rituximab cannot (38, 97). The newer irreversible proteasome inhibitor carfilzomib offers greater selectivity and fewer peripheral neuropathic side effects, and has been explored for desensitization in sensitized recipients (102, 103).

#### 4.1.2 Key clinical evidence

The landmark evidence for bortezomib in treating AMR comes from kidney transplantation. Everly et al. first reported in 2008 that a single cycle of bortezomib reduced DSA levels and improved graft function in patients with refractory mixed rejection (97). Woodle et al. subsequently used an iterative design to systematically evaluate bortezomib−containing desensitization/treatment regimens (101). However, not all studies have been consistent: Sberro−Soussan et al. found that bortezomib alone as the sole desensitization modality did not reduce DSA levels in sensitized kidney transplant recipients, suggesting that its efficacy is highly dependent on combination with plasma exchange and IVIG, as well as on timing of administration (104). Evidence in the heart transplantation setting is more limited and fragmentary, consisting mostly of single−center case series and individual case reports. For example, Morrow et al. reported that bortezomib achieved rapid DSA reduction and reversal of AMR in pediatric heart transplant recipients (105). In adult patients with refractory AMR, bortezomib is typically used as a salvage option after failure of plasma exchange/IVIG/rituximab, and is often administered in combination with these agents, making it difficult to attribute efficacy to the drug alone (10, 31, 106) (**Table 2**). Regarding safety, peripheral neuropathy is the most characteristic adverse effect (mostly grade 1–2, dose−related, reversible), followed by thrombocytopenia, gastrointestinal reactions, and an increased risk of opportunistic infections (97, 102, 107–111).

**Table 2:** Summary of principal reports of bortezomib for antibody-mediated rejection in heart transplantation.

Author/year	Design	n	AMR type	Combination	DSA change	Cardiac function	Neuropathy	Main conclusion
Morrow 2012	Case series	4	Acute	PLEX + IVIG ± RTX	Rapid DSA ↓	Improved/stabilized	Not reported	Bortezomib reversed AMR and reduced DSA in pediatric HTx
Patel 2011	Case series	6	Pre-transplant desensitization	PLEX + IVIG	DSA ↓	Variable	Reported	Proteasome inhibition reduces alloantibody in cardiac Tx
Eckman (case)	Case report	1	Refractory	Multimodal	DSA ↓	Improved	Mild	Adjunctive benefit in refractory cardiac AMR
Multi-organ series (Everly/Woodle, incl. heart)	Cohort	Predominantly kidney; few heart	Mixed	PLEX + RTX	≈50% with >50% DSA ↓	—	Reported	Effect confounded by concomitant therapy

PLEX, plasma exchange; RTX, rituximab. There is no dedicated randomized controlled trial of bortezomib in cardiac AMR; all evidence derives from observational, frequently combination-confounded reports. Patient numbers are those reported in the cited primary studies.

#### 4.1.3 Controversies regarding timing of use and combination regimens

Regarding when to initiate a proteasome inhibitor, the current clinical consensus is generally as follows: for refractory AMR that does not respond to first−line therapy, or for early intervention in high−risk situations characterized by high−MFI DSA with hemodynamic compromise (10, 31). In terms of sequencing, a physiologically rational approach is to “clear circulating antibodies with plasma exchange first, then inhibit antibody re−production with bortezomib, supplemented by IVIG for modulation” — that is, a sequential PLEX → bortezomib → IVIG strategy. However, the specific number of cycles, dosing intervals, and whether to add rituximab vary considerably among centers, and no high−quality evidence supports any fixed regimen (10, 31, 80).

### 4.2 Blocking the terminal complement pathway: C5 inhibitors

#### 4.2.1 Pharmacological mechanism

Eculizumab is a humanized monoclonal antibody that binds C5 and blocks its cleavage into C5a and C5b−9. While inhibiting terminal complement effects (pro−inflammatory C5a and cytolytic MAC), it preserves opsonization and immune complex clearance at the C3 level (41, 50). Mechanistically, this “site−specific precision blockade” precisely corresponds to the core complement−dependent injury pathway in AMR (47, 50).

#### 4.2.2 Clinical evidence

The strongest evidence comes from perioperative prevention in sensitized recipients. In kidney transplantation, Stegall et al. demonstrated that terminal complement inhibition significantly reduced the incidence of early AMR in sensitized recipients (112); subsequently, Marks et al. conducted a randomized trial further evaluating the preventive value of eculizumab in desensitization for living donor kidney transplantation (113). In heart transplantation, a study by Patel et al. in immunologically high−risk recipients suggested that perioperative use of eculizumab reduced early AMR (41); retrospective experiences from multiple centers also indicated that among highly sensitized recipients with cPRA >70% and pre−existing DSA but negative CDC crossmatch, a perioperative eculizumab regimen achieved a favorable one−year survival rate without hyperacute rejection (114). Evidence for treating established acute AMR is weaker: case series suggest that eculizumab rapidly inhibits complement−mediated injury and stabilizes hemodynamics, but it has no clearing effect on DSA itself, and recurrence risk exists after discontinuation. Therefore, it is better positioned as a bridging/salvage measure for severe acute AMR rather than a curative therapy (115, 116); there are also case reports of its use as a bridge to retransplantation in chronic AMR (117–120).

#### 4.2.3 Practical challenges

The clinical application of eculizumab faces three major constraints. First, terminal complement blockade significantly increases the risk of Neisseria (especially meningococcal) infections; vaccination against meningococcus is mandatory before use, and prophylactic antibiotics should be considered when necessary (41, 107). Second, the high cost imposes a heavy economic burden for long−term use — this “financial toxicity” directly affects accessibility across different healthcare systems (31, 121). Third — and most fundamentally from a mechanistic perspective — it cannot block complement−independent ADCC injury, which at the molecular level defines the upper limit of efficacy of C5 inhibitors (25, 56). The long−acting C5 monoclonal antibody ravulizumab reduces dosing frequency and is being explored in the transplantation field, but it remains subject to the same ADCC−related ceiling (122, 123).

### 4.3 Upstream regulation of antibody production: IL−6 pathway inhibitors

#### 4.3.1 Dual−action potential

IL−6 is both a key cytokine driving B−cell differentiation into plasma cells and promoting antibody production, and a central mediator of vascular endothelial activation and local inflammation. Therefore, blocking the IL−6/IL−6R axis theoretically confers a “double dividend”: reducing DSA production upstream while directly mitigating inflammatory endothelial injury in AMR. This makes it particularly attractive in chronic AMR and CAV progression, which are characterized by chronic microvascular inflammation (42, 124–126).

#### 4.3.2 Clinical evidence

Early evidence for the anti−IL−6R monoclonal antibody tocilizumab came primarily from HLA−highly sensitized kidney transplant recipients. Choi et al. reported that tocilizumab stabilized graft function and reduced DSA in refractory chronic AMR and transplant glomerulopathy (124); Vo et al. used it for desensitization in patients who had failed conventional desensitization (127); Pottebaum et al. reported on the efficacy and safety of tocilizumab for active acute AMR (128). Data in the heart transplantation field are still accumulating, mostly consisting of case reports incorporating tocilizumab into multi−drug salvage regimens (117). The anti−IL−6 monoclonal antibody clazakizumab has a higher level of evidence: a phase II study Doberer et al. suggested that it modulates DSA, stabilizes renal function, and has a manageable safety profile (42); the subsequent phase III IMAGINE trial (NCT03744910) was the largest placebo−controlled study in chronic active AMR to date (129). However, its results delivered a sobering message to the entire field: an interim analysis based on the one−year eGFR slope indicated that the trial was unlikely to meet its primary endpoint, leading to early termination (130). The clinical implications of this negative result are twofold — it warns against overestimating the ability of a single upstream target to reverse established chronic injury, and it highlights that “disconnect between surrogate endpoints (eGFR slope) and hard endpoints” is a real pitfall in the design of such trials (129–132).

#### 4.3.3 Implications for heart transplantation

Although IMAGINE did not meet its primary endpoint in chronic AMR of kidney transplantation, the value of IL−6 pathway blockade in heart transplantation should not be dismissed outright. The injury dynamics and endpoint definitions differ between cardiac and renal AMR, and IL−6 inhibition may hold greater potential for early, active, inflammation−predominant AMR. The heart transplantation field should closely follow this “spillover evidence” from other organs and carefully design prospective studies targeting active AMR (31, 125, 130) (**Table 3**).

**Table 3:** Eculizumab versus tocilizumab in cardiac AMR: an indirect comparison.

Feature	Eculizumab (anti-C5)	Tocilizumab (anti-IL-6R)
Step in pathway	Terminal complement (downstream effector)	IL-6 signaling (upstream antibody generation + inflammation)
Effect on DSA	None (blocks effect, not production)	Reduces DSA production over time
Representative studies	Stegall, Marks, Patel, Kittleson	Choi, Vo, Pottebaum
Best-suited scenario	Acute, complement-driven (C4d+); peri-operative prevention in sensitized	Chronic/active inflammatory AMR; transplant glomerulopathy
Onset of action	Rapid (hours–days)	Slower (weeks)
Relapse after discontinuation	High (DSA unaffected)	Lower (modifies DSA), but rebound reported
Infection signature	Neisseria/meningococcal (vaccination mandatory)	General bacterial; GI perforation risk; masks CRP
Relative cost	Very high	High (biosimilars emerging)
Key mechanistic ceiling	Cannot block complement-independent ADCC	Does not rapidly clear established antibody

ADCC, antibody-dependent cellular cytotoxicity; CRP, C-reactive protein; DSA, donor-specific antibody. No head-to-head randomized comparison exists; this table is an indirect, mechanism-based comparison intended to guide phenotype-matched selection.

### 4.4 Other noteworthy strategies

#### 4.4.1 Anti−CD38 monoclonal antibodies: more complete plasma cell depletion

CD38 is highly expressed on the surface of plasma cells. The anti−CD38 monoclonal antibody daratumumab can therefore deplete antibody−secreting cells more directly than bortezomib, while also acting on CD38+ NK cells and plasmablasts (133, 134). Encouraging case reports and small−series data exist for refractory AMR in heart transplantation: after failure of conventional and bortezomib−based regimens, addition of daratumumab resulted in decreased DSA titers, conversion of dd−cfDNA to negative, and improvements in pAMR grade and hemodynamics (135, 136). Of greater methodological significance, the next−generation anti−CD38 monoclonal antibody felzartamab has completed a randomized, double−blind, placebo−controlled phase II trial in late−stage AMR of kidney transplantation, demonstrating improvement in molecular markers of AMR activity — providing the highest−level evidence to date for the anti−CD38 strategy (137). Its regulatory status has since advanced beyond the purely investigational: as of 2025 felzartamab holds FDA Breakthrough Therapy Designation for late AMR without concurrent T-cell-mediated rejection and Orphan Drug Designation for AMR in kidney transplantation, and a pivotal phase III trial (TRANSCEND, NCT06685757) is under way (137, 138). This class of agents holds promise, but careful attention must be paid to the risk of infection and the impact on protective antibodies (134, 137, 139–147).

#### 4.4.2 Co−stimulation blockade

Belatacept inhibits T−cell activation by blocking CD28−B7 co−stimulation, thereby indirectly attenuating T−cell−dependent antibody responses (148). Long−term data from kidney transplantation (e.g., the BENEFIT series) show improved renal function and reduced *de novo* DSA formation, but with an increased risk of cellular rejection (148, 149). In heart transplantation AMR, the role of belatacept remains unclear; its potential role is more likely in preventing *de novo* DSA than in treating established acute AMR (31, 149–153). Although most belatacept data derive from kidney transplantation, cardiac-specific evidence has begun to appear. A recent propensity-matched heart transplant cohort described a belatacept-based, calcineurin-inhibitor-sparing regimen and reported preservation of renal function together with acceptable rejection and survival outcomes and a signal toward reduced *de novo* DSA (154). In the desensitization setting, a proof-of-concept case series combined belatacept with proteasome inhibition and achieved marked reductions in class I and II HLA antibodies and negative complement-dependent cytotoxicity crossmatches in highly sensitized heart transplant candidates (155). These reports remain small and hypothesis-generating, but they indicate that belatacept is being actively evaluated in the cardiac context rather than solely in the kidney.

#### 4.4.3 Emerging complement inhibitors

Beyond eculizumab, multiple nodes in the complement cascade are being developed as therapeutic targets. C1 esterase inhibitor blocks the initiation of the classical pathway and has been explored in desensitization of sensitized recipients and in kidney transplant AMR (156, 157). The anti−C1s monoclonal antibody sutimlimab has completed a phase Ib study in late−stage AMR (158). The C3 inhibitor pegcetacoplan theoretically blocks the generation of C3a, C5a, and MAC simultaneously, providing more upstream and broader complement blockade than C5 inhibition (159). The common value of these agents lies in offering the possibility of “choosing the depth of blockade on demand” at different levels of the complement cascade. However, they similarly cannot reach complement−independent ADCC pathways (56, 159, 160).

#### 4.4.4 New tools for antibody clearance and neutralization

The immunoglobulin−degrading enzyme imlifidase (IdeS) cleaves circulating IgG (including DSA) within hours, creating a window for transplantation in highly sensitized recipients. It has demonstrated rapid DSA clearance in desensitization, but its effect is transient and does not suppress antibody re−production (161). Antagonists targeting FcRn lower DSA levels by accelerating IgG catabolism, representing a new “non−depleting antibody−lowering” approach; their evaluation in transplantation is still in its early stages (162, 163) (**Table 4**).

**Table 4:** Selected key clinical trials of targeted therapy relevant to AMR (heart and kidney).

Trial/study	Registration	Drug (target)	Population	Primary endpoint	Status	Relevance to heart Tx
IMAGINE	NCT03744910	Clazakizumab (IL-6)	Kidney, chronic active AMR	Time to all-cause graft loss (eGFR-slope surrogate)	Terminated early; did not meet primary endpoint	Tempers expectations for IL-6 blockade in chronic AMR
Eculizumab in sensitized HTx	NCT02013037	Eculizumab (C5)	Highly sensitized heart recipients	≥pAMR2 and/or LV dysfunction at 1 yr	Completed (single-arm)	Direct heart-transplant evidence for peri-operative C5 blockade
Felzartamab AMR	NCT05021484	Felzartamab (CD38)	Kidney, late AMR	Safety; molecular AMR activity	Phase 2 reported (2024)	Proof-of-concept for anti-CD38 in AMR
INTERCEPT (tocilizumab)	NCT04561986	Tocilizumab (IL-6R)	Kidney, chronic active AMR	DSA/graft function	Ongoing (RCT)	Spillover rationale for IL-6R blockade
TRANSCEND (felzartamab)	NCT06685757	Felzartamab (CD38)	Kidney, late AMR	Composite graft outcome	Phase 3, recruiting	Pivotal anti-CD38 trial; nearest to practice-changing AMR evidence

eGFR, estimated glomerular filtration rate; HTx, heart transplantation; LV, left ventricular. All registration numbers were verified on ClinicalTrials.gov.

## 5 From single agents to regimens: integration and precision in AMR therapy

**Figure 2:**
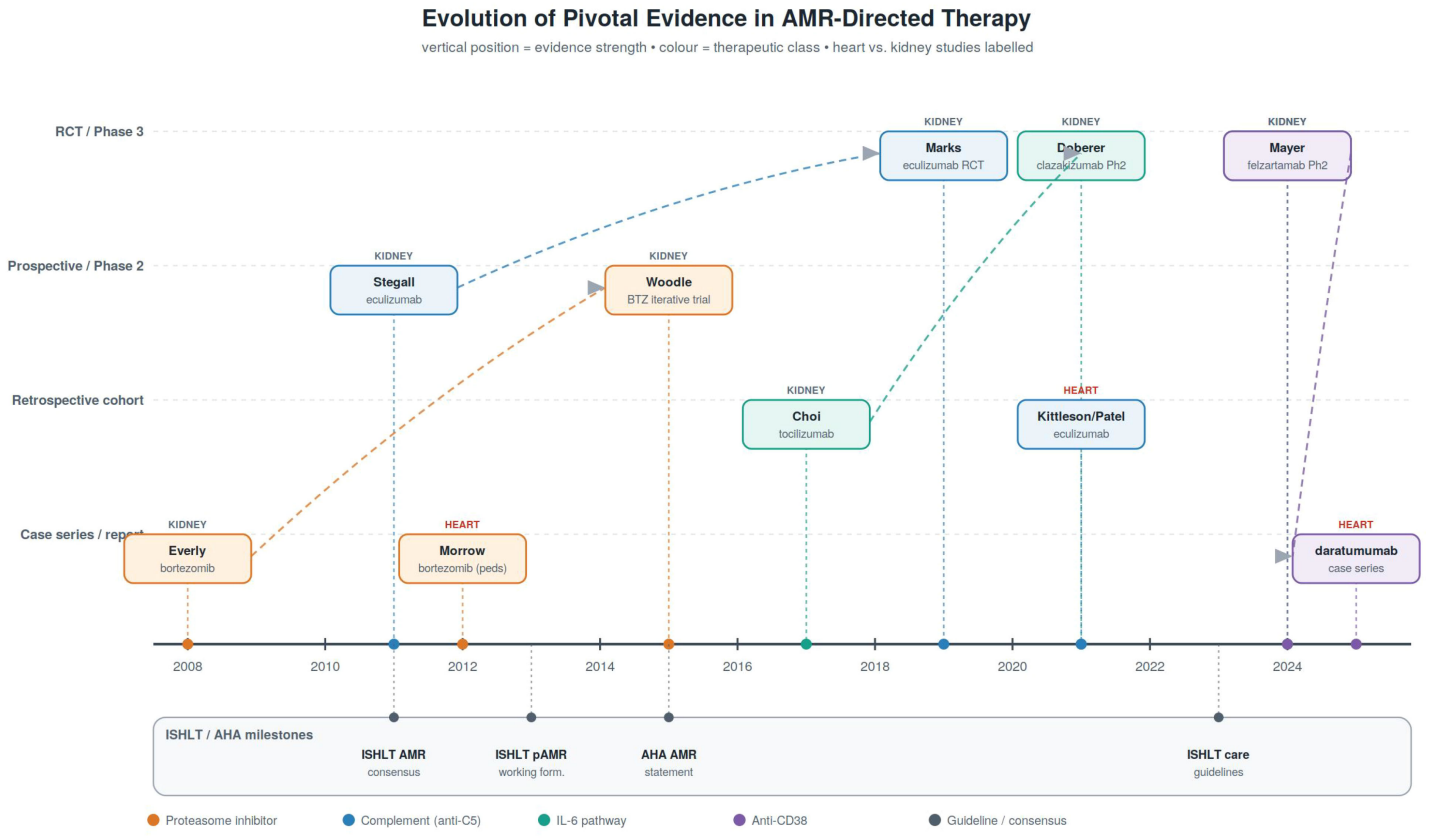
Evolution of pivotal evidence in AMR-directed therapy. Vertical position reflects evidence strength (case report/series to retrospective cohort to prospective/phase 2 to RCT/phase 3); color denotes therapeutic class; heart and kidney studies are labelled. Curved dashed arrows indicate representative translational trajectories. ISHLT/AHA consensus and guideline milestones are marked along the lower axis.

The continuous emergence of novel agents has not automatically translated into improved clinical outcomes — on the contrary, the increasing number of “weapons” has magnified the challenge of “how to deploy them.” This section attempts to integrate the scattered tools into an actionable strategic framework (**Figure 2**).

### 5.1 Constructing a stratified treatment strategy

The intensity of AMR treatment should be determined by three dimensions collectively, rather than by a “one−size−fits−all” approach. The first is clinical severity: subclinical AMR (pathology plus DSA only, with normal cardiac function), acute symptomatic AMR (accompanied by decreased cardiac function), and severe AMR with hemodynamic compromise/cardiogenic shock — the urgency of treatment differs markedly among these three categories (3, 4, 31). The second dimension is DSA characteristics: including MFI level, complement−fixing capacity (e.g., C1q−binding positivity indicates stronger pathogenicity), DSA number, class (class I/II), and epitope specificity (26, 164, 165). The third dimension is pathological features: C4d positivity suggests a complement−dependent pathway predominance, whereas C4d negativity with predominant macrophage infiltration suggests ADCC — the expected response to complement inhibitors differs between these two patterns. The degree of microvascular inflammation and the presence or absence of myocyte necrosis also directly inform decisions to escalate therapy (21, 25, 56, 166–168).

**Figure 3:**
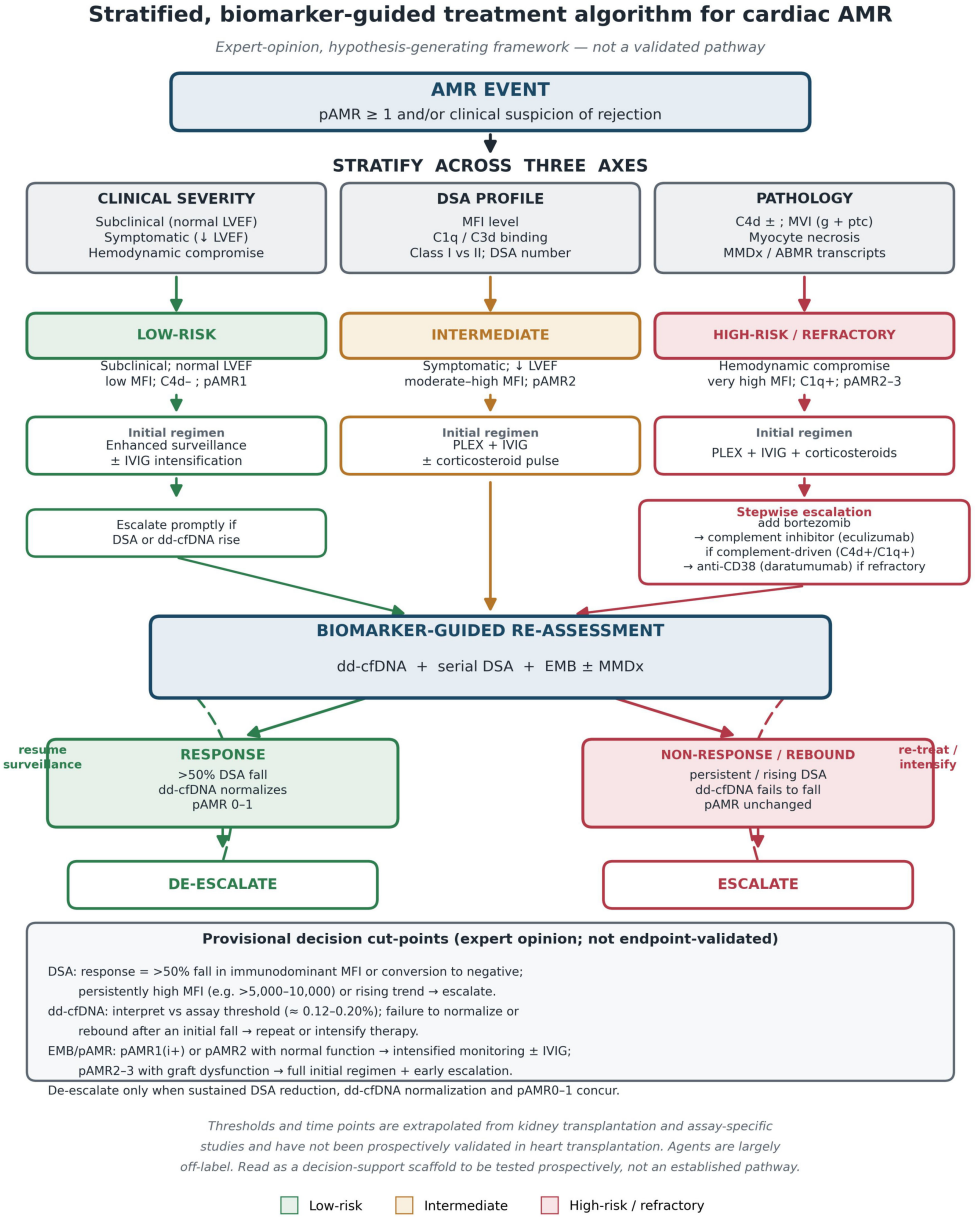
Proposed stratified, biomarker-guided treatment algorithm for cardiac AMR. Cases are stratified across three axes (clinical severity, DSA profile, pathology) into low-risk, intermediate, and high-risk/refractory groups, each linked to an initial regimen. dd-cfDNA, serial DSA, and EMB +/- MMDx drive a biomarker-guided escalation/de-escalation loop. This algorithm is presented as an expert-opinion, hypothesis-generating framework rather than a clinically validated pathway; the thresholds and time points are extrapolated from kidney transplantation and assay-specific studies and have not been prospectively validated in heart transplantation.

Based on these considerations, a stratified pathway can be outlined (see **Figure 3** decision tree). For subclinical/low−risk patients, enhanced monitoring is the mainstay, with IVIG intensification when DSA levels rise. For acute symptomatic patients, plasma exchange plus IVIG ± corticosteroid pulses are used as the initial regimen. For refractory or high−risk cases (high MFI, hemodynamic compromise), bortezomib is added to the above foundation, with optional combination of eculizumab depending on evidence of complement activation, and escalation to anti−CD38 monoclonal antibody if necessary (10, 31). This pathway is not a rigid dogma but an “escalation/de−escalation” framework that adjusts dynamically according to biomarkers. Because the choice of agent is the step clinicians find most difficult, we make it explicit here: **Table 5** pairs each dominant AMR phenotype with a potential initial option, framed as an expert-opinion-based approach rather than an evidence-graded recommendation, together with the reason for that choice and its principal caveat.

**Table 5:** When to use which agent: a phenotype-based, expert-opinion guide to initial agent selection in cardiac AMR.

AMR phenotype	Potential initial option (expert-opinion-based)	Rationale	Key caveat
Acute, complement-driven (C4d-positive and/or C1q-binding DSA; hemodynamic compromise)	PLEX + IVIG, plus a terminal complement inhibitor (eculizumab)	Rapidly interrupts C5a- and MAC-mediated endothelial injury at the dominant effector step	Does not clear DSA; meningococcal vaccination mandatory; high cost; relapse after withdrawal
Acute, C4d-negative (NK-cell/macrophage-predominant microvascular inflammation)	PLEX + IVIG, plus plasma-cell-directed therapy (bortezomib ± daratumumab)	Injury is largely ADCC-driven, so complement blockade alone is mechanistically insufficient	Anti-CD38 interferes with HLA crossmatch; cytopenias; C5 inhibition of limited value here
Refractory acute (inadequate response to PLEX/IVIG ± rituximab)	Proteasome inhibitor (bortezomib), escalating to anti-CD38 (daratumumab)	Reaches the long-lived plasma cells that sustain DSA and that rituximab cannot deplete	Peripheral neuropathy (bortezomib); added infection risk; evidence mostly from case series
Chronic active, inflammation-predominant (microvascular inflammation, evolving CAV)	IL-6 pathway blockade (tocilizumab) as sustained modulation	Simultaneously dampens endothelial inflammation and upstream antibody production	Slow onset (weeks); established fibrosis is not reversed; IMAGINE missed its primary endpoint
Subclinical/low-risk (pAMR1–2, normal graft function, low MFI)	Enhanced surveillance ± IVIG intensification	Avoids the infectious and financial cost of over-treatment	Escalate promptly if DSA or dd-cfDNA rise

Phenotype-based, expert-opinion guidance intended to make agent selection explicit; it is not validated by randomized trials in heart transplantation, and most agents are used off-label with supporting data extrapolated largely from kidney transplantation. Selection must remain individualized and biomarker-driven (Section 5.2). ADCC, antibody-dependent cellular cytotoxicity; CAV, cardiac allograft vasculopathy; dd-cfDNA, donor-derived cell-free DNA; DSA, donor-specific antibody; IVIG, intravenous immunoglobulin; MAC, membrane attack complex; MFI, mean fluorescence intensity; PLEX, plasma exchange. Pre-transplant desensitization of highly sensitized candidates is a distinct clinical setting, not an AMR phenotype; it is therefore discussed separately in the text and is not listed as a phenotype row in this table.

### 5.2 Biomarker−driven monitoring and adjustment

Another pillar of precision is shifting from “empirically determined treatment duration” to “evidence−guided intensity adjustment.” Donor−derived cell−free DNA (dd−cfDNA) is among the most promising biomarkers in this regard: its elevation reflects graft injury, can warn of AMR months before histological/clinical diagnosis, and dynamically reflects treatment response. In the daratumumab case series, the decline in dd−cfDNA was highly synchronized with improvement in pAMR grade, and its rebound effectively guided the timing of repeated therapy (136, 169, 170). Dynamic DSA monitoring is equally critical: a >50% reduction in MFI or conversion to negativity indicates treatment efficacy, whereas persistent positivity or an increasing trend signals the need for escalation (26, 164). Furthermore, AlloMap (peripheral blood gene−expression profiling) and the molecular diagnostic system based on biopsy tissue (Molecular Microscope, MMDx) have unique value in distinguishing rejection types and identifying C4d−negative AMR, complementing traditional histology (171–173). We advocate constructing an integrated triple−dynamic monitoring strategy combining “endomyocardial biopsy + dd−cfDNA + DSA”: using dd−cfDNA as a highly sensitive early−warning radar, DSA to reflect immune burden, and biopsy (supplemented by MMDx when necessary) as the gold standard for diagnosis and grading. These three modalities corroborate one another and jointly drive treatment escalation or de−escalation (169, 171, 173–186).

To make the preceding framework actionable rather than merely conceptual, we propose the following provisional thresholds and time points; they are offered as an expert-opinion, hypothesis-generating scheme, are extrapolated from kidney data and from assay-specific cardiac studies, and have not been prospectively validated as a treatment pathway in heart transplantation. DSA: quantify at diagnosis and then every 1–2 weeks during active treatment, monthly to month 3–6, and thereafter per surveillance; a response is defined as a >50% fall in immunodominant-DSA MFI or conversion to negative, whereas a persistently high immunodominant MFI (for example >5,000–10,000 on a single-antigen-bead assay) or a rising trend argues for escalation. Complement-binding DSA (C1q or C3d positivity): when present, prioritize complement-directed therapy and closer monitoring. dd-cfDNA: interpret against the assay-specific threshold (commonly a fraction of about 0.12–0.20% for the widely used assays); failure to normalize, or a rebound after an initial fall, supports repeating or intensifying therapy. EMB/pAMR: pAMR1(i+) or pAMR2 with normal graft function warrants intensified monitoring with or without IVIG, whereas pAMR2–pAMR3 accompanied by graft dysfunction warrants full first-line therapy with early targeted escalation. Graft dysfunction: any new fall in left ventricular ejection fraction or hemodynamic compromise reclassifies the episode as high-risk/severe irrespective of the other axes. De-escalation is considered only when a sustained DSA reduction, normalization of dd-cfDNA, and pAMR0–1 on a follow-up biopsy are concordant. Because none of these cut-points has been validated against hard cardiac endpoints, the algorithm in **Figure 3** should be read as a decision-support scaffold to be tested prospectively, not as a clinically established pathway (26, 136, 164, 169, 170).

### 5.3 The “full−chain blockade” theory of combination therapy

Mapping mechanisms onto drugs yields an intuitive theoretical model of combination therapy: inhibit antibody production upstream (IL−6 inhibitors, proteasome inhibitors, anti−CD38 monoclonal antibodies), clear circulating antibodies in the middle stream (plasma exchange, imlifidase), and block effector injury downstream (complement inhibitors, IVIG) (31, 159). A representative aggressive regimen covering the full chain might be a sequential or concurrent combination of “plasma exchange + bortezomib + IVIG + eculizumab,” with the course guided by dd−cfDNA/DSA (10, 31). However, it must be clearly stated that this “full−chain blockade” remains a theoretical advantage at the mechanistic level only. No randomized controlled evidence has yet demonstrated that multi−target combination is superior to existing empirical combinations for hard endpoints, whereas the additive infectious risk and economic burden of combination therapy are certain (31, 121). Therefore, the “addition” of combination therapy must be predicated on stratification and biomarkers, achieving “add when indicated, de−escalate when possible.”

## 6 Current challenges and practical dilemmas

### 6.1 Structural dilemma of evidence level

The persistent lack of large−scale RCTs dedicated to AMR in heart transplantation is not accidental but results from several structural characteristics. First, the event rate is relatively low: the annual volume of heart transplant procedures is limited, and only a fraction of these develop clinically significant AMR, making it difficult for a single center to accumulate sufficient hard−endpoint events within a reasonable timeframe (1, 31). Second, patient heterogeneity is substantial: the degree of sensitization, DSA characteristics, AMR phenotype, and the presence or absence of concomitant cellular rejection vary widely, such that “the same type of AMR” scarcely exists (21, 164). Third, endpoint measures are difficult to standardize: surrogate endpoints such as DSA reduction or improvement in pAMR grade do not have a simple linear correspondence with hard endpoints such as survival or CAV — the disconnect between the “eGFR slope” surrogate endpoint and hard endpoints in the IMAGINE trial serves as a cautionary example (130, 187). A realistic way to break the deadlock is through multi−center collaborative networks and registry studies: by adopting uniform diagnostic criteria, sharing data, and using regulatorily accepted surrogate endpoints, credible evidence can be generated despite the constraints of rare−disease−level sample sizes (31, 188).

### 6.2 Chronic AMR: still a “therapeutic desert”

If there are still some “weapons” available for acute AMR, chronic AMR is nearly a therapeutic desert. It is characterized by persistent low−grade microvascular inflammation, slowly progressive interstitial fibrosis, and microvascular rarefaction, and clinically often presents as insidious graft dysfunction that eventually converges into CAV (11, 12, 70). The essence of the problem is that most existing targeted agents act on the “production” and “acute effector” phases of antibodies and have little effect on established fibrosis and vascular remodeling. The failure of IMAGINE to meet its primary endpoint in chronic active AMR to some extent confirms the harsh reality that “established structural injury is difficult to reverse by immunological suppression alone” (70, 130). Therefore, potential breakthroughs lie not in “stronger immunosuppression” but in three directions: moving the battlefront forward to the subclinical, active stage to intervene before fibrosis develops; exploring the potential value of anti−fibrotic agents in graft fibrosis; and evaluating the long−term, low−intensity modulatory effects of IL−6 pathway blockade on chronic inflammation (32, 70, 125).

### 6.3 Combination therapy: ideal versus reality

**Figure 4:**
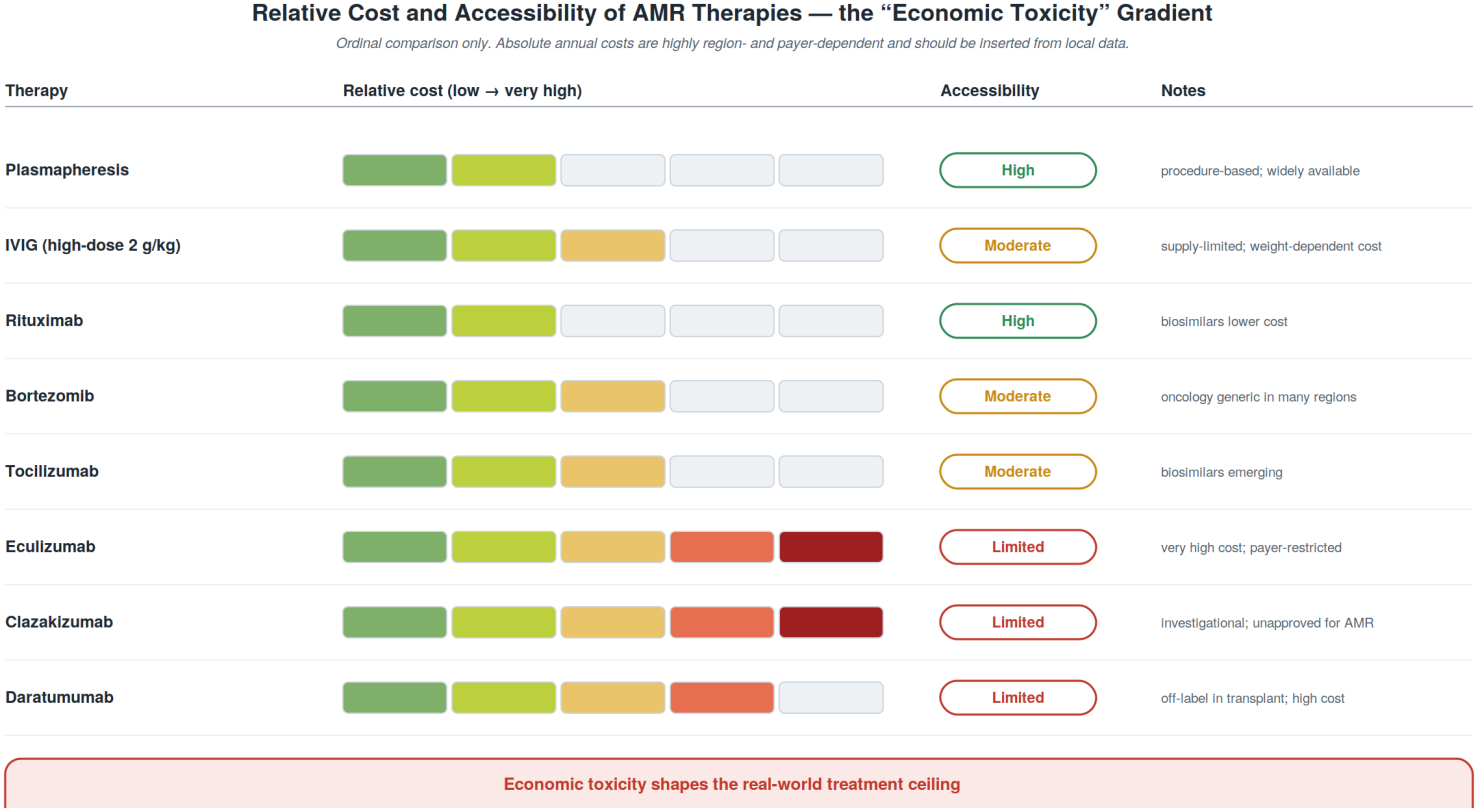
Relative cost and accessibility of AMR therapies (the economic-toxicity gradient). Ordinal comparison only; absolute annual costs are highly region- and payer-dependent and should be inserted from local data before submission. The most mechanistically attractive agents (terminal-complement and anti-CD38 antibodies) cluster at the high-cost/low-accessibility end, such that reimbursement policy, rather than biology, frequently determines the achievable regimen.

The concept of “full−chain blockade” is mechanistically appealing, but its clinical implementation faces two certain costs. The first is the additive infectious risk: intensified humoral immunosuppression exposes recipients to multiple opportunistic infections — cytomegalovirus, BK virus, Epstein−Barr virus−related lymphoproliferative disease, Pneumocystis jirovecii pneumonia — and terminal complement inhibition specifically adds the risk of Neisseria (meningococcal) infection (41, 106, 107, 189). It must be emphasized that the infectious risk of combination therapy is not merely the sum of the risks of each individual agent — synergism of immunosuppression often leads to a non−linear amplification of the overall risk, necessitating more proactive prophylaxis and intensified microbiological surveillance (189, 190) (**Table 6**). The second cost is financial toxicity: biologic agents such as eculizumab, daratumumab, and clazakizumab carry high annual treatment costs, imposing a heavy burden under most healthcare systems. Substantial variation in reimbursement policies among different countries and regions often creates a gap between the “theoretically optimal regimen” and the “actually accessible regimen” for patients — this practical constraint must be incorporated into clinical decision−making (31, 121, 191) (**Figure 4**).

**Table 6:** Infection risk and management considerations by AMR treatment modality.

Modality	Predominant infection risk	Notable pathogens	Prophylaxis	Monitoring
Plasmapheresis	Catheter-related; transient hypogammaglobulinemia	Bacterial (line sepsis)	Aseptic line care	Cultures if febrile
IVIG	Low (may be protective)	—	—	—
Rituximab	Viral/bacterial; late hypogammaglobulinemia	HBV reactivation, PML (rare)	HBV screening; consider PJP cover	IgG levels, HBV
Bortezomib	Viral reactivation	VZV, CMV	Antiviral (VZV) prophylaxis	CMV PCR
Eculizumab	Encapsulated organisms	Neisseria meningitidis (and gonococcus)	Meningococcal vaccination (mandatory) ± antibiotic prophylaxis	Clinical vigilance for meningococcemia
Tocilizumab/Clazakizumab	Bacterial; GI	Diverticulitis/perforation; masks fever/CRP	PJP cover per program	Lipids, LFTs, neutrophils
Daratumumab	Broad (plasma-cell depletion)	CMV, PJP, hypogammaglobulinemia-related	PJP and antiviral prophylaxis; IVIG replacement if low IgG	IgG, CMV PCR; note interference with HLA crossmatch

CMV, cytomegalovirus; CRP, C-reactive protein; HBV, hepatitis B virus; LFT, liver function test; PJP, Pneumocystis jirovecii pneumonia; PML, progressive multifocal leukoencephalopathy; VZV, varicella-zoster virus. Infection risk under combination therapy is supra-additive rather than simply additive and warrants heightened, protocolized surveillance.

### 6.4 Limitations of diagnostic and monitoring tools

Precision therapy presupposes precision diagnosis, yet the current toolkit still has notable shortcomings. Endomyocardial biopsy, as the gold standard, suffers from sampling error, limited inter−observer agreement, risk of complications, and difficulty in frequent repetition (21, 192). C4d staining is influenced by fixation methods and interpretive experience, and is inherently “blind” to C4d−negative AMR (21, 25). dd−cfDNA has high sensitivity but limited specificity due to interference from various non−rejection forms of injury; standardization of thresholds and multi−center validation are still ongoing (169, 170, 193). Molecular diagnostics (MMDx), although able to complement histological limitations, are costly and have limited accessibility, and have not yet been widely incorporated into routine clinical practice (171, 173, 194).

## 7 Future directions and potential breakthroughs

**Figure 5:**
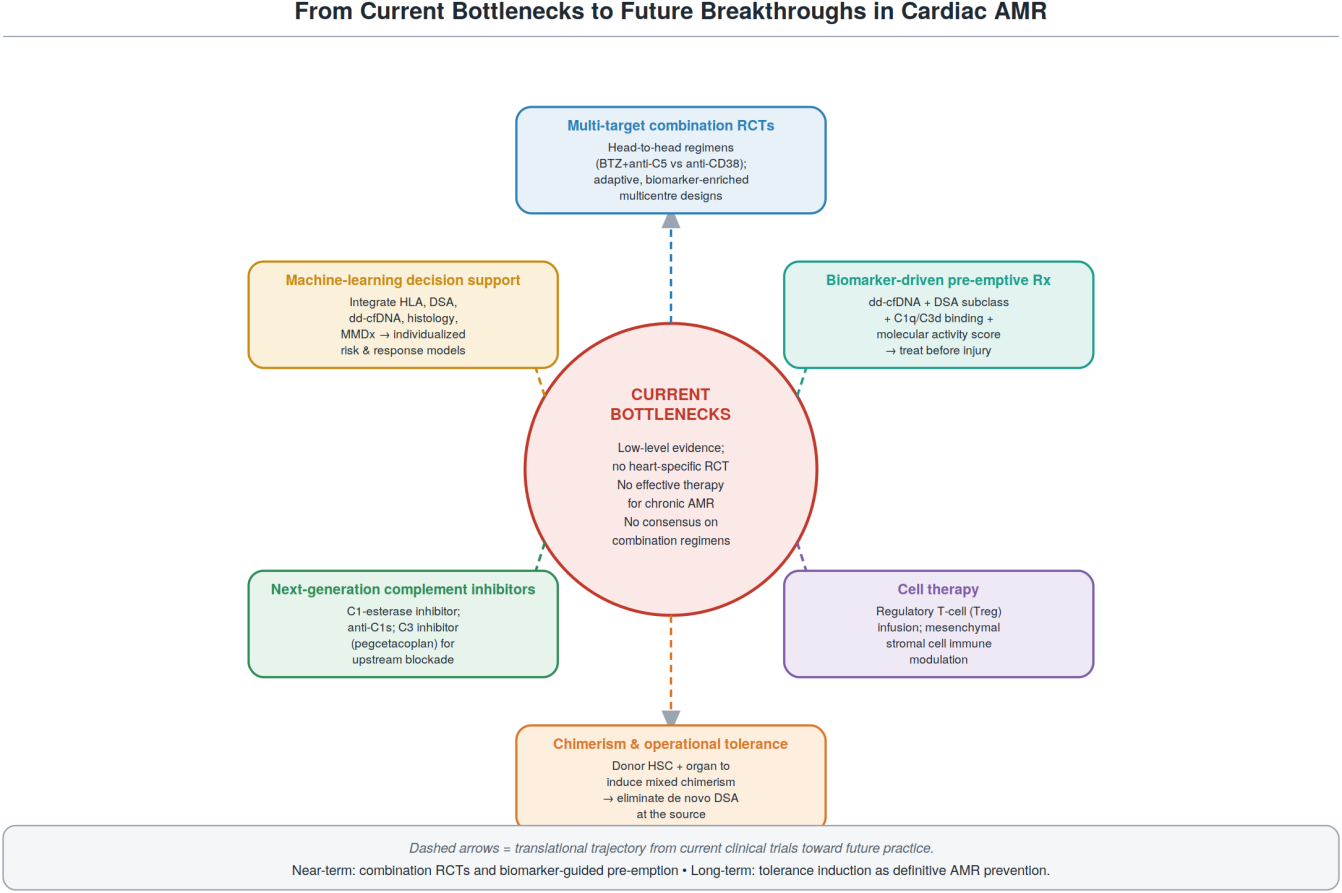
From current bottlenecks to future breakthroughs in cardiac AMR. The central hub summarizes present bottlenecks (low-level evidence with no heart-specific RCT, no effective therapy for chronic AMR, no consensus on combinations). Spokes denote breakthrough directions: multi-target combination RCTs, biomarker-driven pre-emptive therapy, cell therapy, chimerism and operational tolerance, next-generation complement inhibitors, and machine-learning decision support. Dashed arrows represent the translational trajectory from current trials toward future practice.

The next phase of AMR therapy will not merely be about “adding another weapon,” but will focus on “how to intervene earlier, more precisely, and more sustainably” (see **Figure 5** roadmap).

### 7.1 Prospective trials of multi−target combinations

The most urgent and realistic step is to test the currently mechanism−derived combination regimens in rigorously designed prospective trials — especially head−to−head comparisons of different combinations (e.g., proteasome inhibitor + C5 inhibitor vs. anti−CD38 antibody−based regimens), as well as adaptive trial designs guided by biomarker stratification. Leveraging multi−center networks, unified pAMR and DSA assessment criteria, and composite surrogate endpoints accepted through regulatory discussion, such trials have the potential to generate generalizable conclusions despite the constraints of rare−disease−level sample sizes (31, 137, 188).

### 7.2 Biomarker−driven preemptive therapy

A future paradigm may shift from “treatment after diagnosis” to “preemptive intervention after warning”: early elevation of dd−cfDNA, DSA subclass and complement−binding characteristics, and molecular diagnostic activity scores together constitute risk signals, enabling initiation or escalation of therapy before irreversible damage occurs (136, 169, 170). Among these, fine−typing of DSA subclasses (e.g., IgG3, a strong complement−fixing subclass) and C1q/C3d binding capacity are expected to become key tools for selecting patients most likely to benefit from complement inhibition (164, 165).

### 7.3 Cell therapy and immunomodulation

Cell therapy represents an alternative approach — not relying on “suppression” but on “remodeling” immune balance. Regulatory T cell (Treg) infusion aims to re−establish donor−specific immune tolerance and has shown safety and potential benefit in early−phase trials in kidney and liver transplantation (195, 196). Mesenchymal stem cells exert immunomodulatory effects through multiple paracrine mechanisms and are being explored in solid organ transplantation (197–200). Although these strategies remain at the frontier in heart transplantation AMR, their direction of “inducing tolerance rather than long−term immunosuppression” directly addresses the root of the infection−toxicity dilemma inherent in combination therapy.

### 7.4 Induction of chimerism and operational tolerance: ultimate prevention of AMR

From a longer−term perspective, the highest goal of AMR therapy is to prevent its occurrence altogether. The establishment of mixed chimerism through combined donor hematopoietic stem cell and solid organ transplantation, thereby inducing donor−specific operational tolerance, has achieved proof−of−concept for immunosuppression withdrawal in some kidney transplant recipients (201, 202). If this strategy could be safely extended to heart transplantation, it could theoretically eliminate *de novo* DSA production at the source. Nevertheless, the conditioning toxicity, risk of graft−versus−host disease, and the unique time−window constraints of heart transplantation pose major challenges to its translation into the cardiac setting (202–205).

### 7.5 Computational and artificial intelligence−driven decision support

AMR, characterized by high heterogeneity and numerous variables, is well−suited for machine learning applications. Integrating HLA matching, DSA dynamics, dd−cfDNA, histology and molecular diagnostics, medication history, and outcome data may enable the development of individualized risk prediction and treatment response models, assisting clinicians in making better decisions regarding “when to escalate, which combination to use, and when to de−escalate” (206–208). The value of such tools lies not in replacing clinical judgment, but in transforming dispersed multi−modal evidence into interpretable, bedside−usable decision support.

## 8 Conclusion

The treatment of AMR in heart transplantation has advanced from the empirical era relying solely on plasma exchange and IVIG into an era in which targeted interventions can be directed against multiple molecular nodes, including B cells, plasma cells, terminal complement, and IL−6. Proteasome inhibitors have reached the source of antibody production, complement inhibitors precisely block terminal effector pathways, and anti−CD38 and anti−IL−6 agents have further expanded the boundaries of upstream regulation — our “weapons” are unprecedentedly abundant.

However, abundance of weapons does not equate to victory in the battle. The field remains clearly in a transitional phase characterized by “weapons available but tactics lacking.” The vast majority of evidence derives from small−sample, single−center, uncontrolled observational studies. Chronic AMR remains a therapeutic desert. Combination regimens, while mechanistically appealing, come at the price of definite infectious and economic costs. And the most fundamental question — how to select, combine, and sequence these weapons according to an individual’s immune phenotype — remains unanswered by any consensus. The failure of the IMAGINE trial in chronic active AMR serves both as a warning and as the most authentic illustration of this transitional phase.

The solution lies in the synergy of three elements: determining treatment intensity through clinical−DSA−pathological three−dimensional stratification; driving escalation/de−escalation through dynamic monitoring of “biopsy + dd−cfDNA + DSA”; covering the entire “production−circulation−effector” chain with mechanism−complementary multi−target combinations; and always advancing the battlefront to the subclinical, active stage. To truly upgrade from “empirical combination” to “biomarker−driven precision intervention,” we need multi−center collaborative prospective evidence, more reliable surrogate endpoints, and a long−term strategy oriented toward tolerance induction. This is both the clinical scientific question that cardiac transplantation immunotherapy must answer in the next decade and the most worthwhile direction for improving long−term recipient survival.
